# Cross-modality fusion with EEG and text for enhanced emotion detection in English writing

**DOI:** 10.3389/fnbot.2024.1529880

**Published:** 2025-01-17

**Authors:** Jing Wang, Ci Zhang

**Affiliations:** ^1^School of Foreign Languages, Henan Polytechnic University, Jiaozuo, China; ^2^College of Foreign Languages, Wenzhou University, Wenzhou, China

**Keywords:** emotion detection, EEG, textual analysis, transformer, cross-modality fusion

## Abstract

**Introduction:**

Emotion detection in written text is critical for applications in human-computer interaction, affective computing, and personalized content recommendation. Traditional approaches to emotion detection primarily leverage textual features, using natural language processing techniques such as sentiment analysis, which, while effective, may miss subtle nuances of emotions. These methods often fall short in recognizing the complex, multimodal nature of human emotions, as they ignore physiological cues that could provide richer emotional insights.

**Methods:**

To address these limitations, this paper proposes Emotion Fusion-Transformer, a cross-modality fusion model that integrates EEG signals and textual data to enhance emotion detection in English writing. By utilizing the Transformer architecture, our model effectively captures contextual relationships within the text while concurrently processing EEG signals to extract underlying emotional states. Specifically, the Emotion Fusion-Transformer first preprocesses EEG data through signal transformation and filtering, followed by feature extraction that complements the textual embeddings. These modalities are fused within a unified Transformer framework, allowing for a holistic view of both the cognitive and physiological dimensions of emotion.

**Results and discussion:**

Experimental results demonstrate that the proposed model significantly outperforms text-only and EEG-only approaches, with improvements in both accuracy and F1-score across diverse emotional categories. This model shows promise for enhancing affective computing applications by bridging the gap between physiological and textual emotion detection, enabling more nuanced and accurate emotion analysis in English writing.

## 1 Introduction

Emotion detection is crucial in fields such as human-computer interaction, mental health monitoring, and sentiment analysis (Xu, 2024). Traditional approaches to emotion detection primarily rely on textual analysis, which captures explicit linguistic cues but often misses nuanced emotional states conveyed by physiological signals. Integrating electroencephalography (EEG) data with textual cues promises a more comprehensive understanding of emotional states, as EEG captures real-time neural responses that can reveal implicit emotional reactions not detectable through text alone (Huang, 2024). The EmotionFusion-Transformer framework aims to harness the complementary strengths of both EEG and textual modalities, offering a deeper and more accurate analysis of emotions in English writing (Nimmi and Janet, 2021). By combining these data sources, EmotionFusion-Transformer not only improves accuracy in detecting complex emotions but also expands the potential applications in personalized learning environments, mental health support tools, and emotionally aware AI systems.

Early approaches to emotion detection relied on symbolic AI and knowledge representation, utilizing handcrafted rules and lexicons to interpret emotional content in text (Kim, 2024). For example, systems used sentiment dictionaries or predefined emotion categories to classify text-based inputs, manually mapping words or phrases to corresponding emotional states (Alvi et al., 2023). While these rule-based methods provided foundational insights, they were often limited by rigid structures and a lack of adaptability to nuanced language use. The symbolic AI approach struggled with handling context-dependent expressions or detecting subtle emotions, which hindered its effectiveness in real-world applications (Babu et al., 2020). To address these limitations, researchers began exploring more adaptive, data-driven methods that could capture the variability and complexity of human emotions in a more flexible manner (Cruz and Balahadia, 2022).

The second phase in emotion detection research shifted toward data-driven approaches, particularly with the advent of machine learning models that leveraged larger datasets for improved accuracy (Singh and Sachan, 2021). Machine learning techniques, such as support vector machines and random forests, allowed for automatic learning of emotion patterns from text data without requiring extensive manual rule-setting (Suleimenova et al., 2022). However, these models predominantly focused on textual information, relying on features like word embeddings or n-grams to infer emotional states (Bakar et al., 2020). Despite significant progress, the reliance on textual data alone limited their ability to capture physiological aspects of emotion, which are crucial for a holistic understanding of affective states. Additionally, while machine learning approaches increased adaptability, they were still constrained by the features provided, often lacking the depth needed to fully capture complex emotional experiences.

With advancements in deep learning, particularly in neural networks and pre-trained models, the third phase introduced powerful tools such as convolutional neural networks (CNNs), recurrent neural networks (RNNs), and transformers that significantly enhanced emotion detection capabilities (Jiang, 2024). These models, especially transformers, allowed for more sophisticated processing of sequential data, making it possible to analyze both text and EEG signals in an integrated manner (N'Diaye et al., 2021). Pre-trained language models like BERT and GPT have shown remarkable proficiency in understanding context-rich text, while EEG-based convolutional models enabled the capture of temporal patterns in neural signals associated with emotional responses. Despite these advancements, challenges remain in effectively fusing multi-modal data, as deep learning models for emotion detection often treat text and EEG independently, missing opportunities for cross-modal interactions that could enhance emotional insight.

To address the limitations of previous methods in effectively merging EEG and textual data, the EmotionFusion-Transformer introduces a novel cross-modality fusion approach. This model leverages transformer architectures specifically designed for multi-modal integration, facilitating deeper interaction between text-based and EEG-based emotional cues (Hernández-Pérez et al., 2024). By incorporating both data types simultaneously, the proposed model can achieve a more nuanced detection of emotions in English writing, overcoming the constraints of single-modality models and enabling a richer, context-aware analysis of emotional states.

- The EmotionFusion-Transformer introduces a new cross-modal fusion mechanism, allowing for the simultaneous processing of EEG and textual data.
- The model is designed to handle diverse scenarios, enhancing its applicability across different emotional contexts and making it highly efficient in real-time emotion analysis.
- Experimental results demonstrate that EmotionFusion-Transformer achieves superior accuracy and robustness in emotion detection compared to single-modality models.

## 2 Related work

### 2.1 Cross-modality fusion for emotion detection

Cross-modality fusion is critical in emotion detection, especially when integrating physiological and textual data (Biswas et al., 2024). Multimodal data fusion, which combines multiple information sources such as electroencephalography (EEG) and text, has gained attention in emotion detection to leverage both the neurophysiological insights from EEG and the contextual insights from text. Studies in this area reveal that physiological signals capture subtle emotional shifts that might not be explicit in language. Therefore, fusion of text with EEG can offer a more comprehensive view of emotional states. Techniques like feature concatenation, cross-modal attention mechanisms, and joint embedding models have been explored to combine EEG and text effectively. These methods aim to address the challenges posed by the heterogeneity and variable temporal resolutions in EEG and text data (Başarslan and Kayaalp, 2020). Feature concatenation, a traditional method in fusion models, is often used for baseline comparisons, where features extracted from EEG and text are aligned and integrated into a single feature vector. While straightforward, this approach often fails to capture nuanced interactions between modalities. To overcome this, research has shifted toward attention-based fusion strategies (Pei, 2024). Cross-modal attention mechanisms focus on selectively emphasizing features from each modality relevant to the emotional state. Such mechanisms allow the model to adaptively assign weights based on the input's content and context, leading to better performance in emotion detection tasks. Another key technique is the joint embedding model, where EEG and text data are projected into a common latent space (Zakaria and Sulaiman, 2024). This approach has shown promise as it facilitates seamless interaction and representation learning across modalities. With advancements in neural architectures, Transformer-based models with modality-specific encoders and shared decoders have been used to learn modality-specific as well as shared features, enhancing the alignment and fusion of multimodal data for emotion detection (Sharma and Ghose, 2023). Recent studies show that cross-modal fusion techniques significantly improve emotion recognition performance. However, there remain challenges such as handling the asynchronous nature of EEG and text signals and mitigating modality-specific noise in fusion processes (Chattu and Sumathi, 2023). Emerging solutions incorporate self-supervised learning, domain adaptation, and modality dropout strategies, which aim to increase model robustness and generalization across different tasks and datasets. Integrating these techniques with advanced fusion architectures holds potential for enhancing the accuracy and applicability of multimodal emotion detection models.

### 2.2 EEG-based emotion detection methods

Electroencephalography (EEG) has become a valuable modality in emotion detection research due to its ability to capture real-time neurophysiological responses. EEG data provides information on emotional arousal, valence, and other affective states through the analysis of brainwave patterns across different frequency bands. Key approaches in EEG-based emotion detection rely on feature extraction from these frequency bands, such as delta, theta, alpha, beta, and gamma, each associated with distinct cognitive and emotional processes. Standard techniques in EEG processing include time-frequency analysis, wavelet transforms, and statistical methods that extract features relevant to emotional states. These features are then classified using machine learning algorithms such as support vector machines (SVMs), decision trees, and more recently, deep learning models. Deep learning techniques, particularly convolutional neural networks (CNNs) and recurrent neural networks (RNNs), have shown notable advancements in EEG-based emotion detection. CNNs have been applied for their ability to learn spatial representations from EEG signal topography, while RNNs, especially long short-term memory (LSTM) networks, are useful for capturing temporal dependencies in sequential EEG data. However, the use of Transformers in EEG processing is emerging as a promising direction, given their ability to model long-range dependencies and manage variable-length sequences, which are common in EEG data (Singh et al., 2020). Despite these advances, EEG-based emotion detection faces several challenges. EEG data is susceptible to noise, particularly from muscle artifacts and external interference, which necessitates careful preprocessing and artifact removal techniques. Moreover, EEG signals are inherently subject-specific, which limits the generalizability of emotion detection models across different individuals. Transfer learning, domain adaptation, and personalized modeling approaches have been explored to address these challenges (Teo et al., 2020). Recent work also incorporates attention mechanisms into EEG models, allowing for dynamic focus on specific channels or time points, thereby improving emotion recognition accuracy. These advancements have contributed to making EEG a reliable source for emotion detection, particularly when combined with other modalities such as text.

### 2.3 Transformer models in multimodal emotion recognition

Transformer-based models have gained prominence in emotion detection due to their capabilities in handling sequential and multimodal data (Polyakova, 2023). Transformers excel at capturing long-range dependencies and contextual relationships within and across modalities, making them highly suitable for tasks involving both text and physiological signals like EEG. The attention mechanism in Transformers enables dynamic feature selection and cross-modal alignment, allowing the model to focus on critical aspects of each modality relevant to emotion detection. In multimodal emotion recognition, Transformers have been applied using modality-specific encoders, where separate encoders process each input modality and a shared decoder or cross-attention mechanism integrates the information from each modality. Multimodal Transformers are often built with cross-attention layers, where one modality's features act as queries, while another's features serve as keys and values (Whissell, 2022). This mechanism allows the model to selectively attend to relevant parts of EEG signals based on contextual cues from text, and vice versa. Such architectures have shown substantial improvement in detecting complex emotions, especially when the emotional cues in one modality are weak but can be complemented by cues in the other. Furthermore, self-attention in Transformers allows for parallel processing, making it feasible to handle the large data volumes and high temporal resolution of EEG data efficiently (Shrestha et al., 2020). Hybrid models, combining CNNs for initial feature extraction from EEG with Transformer layers for fusion and sequence modeling, have demonstrated strong performance in multimodal emotion detection. This setup leverages CNNs for spatial feature learning, followed by Transformers to capture inter-modal and temporal dependencies. Some recent studies explore the integration of pre-trained language models with EEG-based models, using pre-trained language embeddings to enhance the emotion recognition capabilities of multimodal systems. These models benefit from transfer learning, allowing them to leverage rich linguistic knowledge while simultaneously learning from EEG signals. Challenges remain in Transformer-based multimodal models, particularly in terms of computational efficiency and the risk of overfitting due to the high dimensionality of EEG and text features (İşçi, 2023). Techniques such as dimensionality reduction, attention pruning, and modality dropout have been proposed to address these issues. Additionally, Transformers are memory-intensive, especially when applied to high-dimensional multimodal data, which has led to the exploration of more efficient attention mechanisms, such as Linformer and Performer architectures. These efforts aim to make Transformer-based multimodal emotion detection more scalable and applicable to real-world scenarios.

### 2.4 Comparison with related contextual and multimodal models

Recent works have explored various approaches to contextual and multimodal emotion recognition, yet they exhibit limitations that our proposed model addresses. Hierarchical transformer models such as Li et al. (2020) and contextualized emotion tagging approaches like Wang et al. (2020) effectively model local and global dependencies within textual data but fail to incorporate physiological signals, such as EEG, which are critical for capturing non-linguistic emotional cues. Moreover, reasoning-based models (Hu et al., 2021) and sentiment-aware networks (Tu et al., 2022) excel in dialogue context modeling but lack mechanisms to detect sentiment shifts or handle idiomatic expressions, limiting their application in more nuanced emotional contexts. Multimodal systems integrating EEG and textual data (Ghosh et al., 2021; Liu and Fu, 2021) demonstrate the potential of cross-modal fusion but treat modalities as largely independent, missing opportunities to leverage their interactions. Knowledge-enriched frameworks such as Panda et al. (2020) focus on external information for enhanced understanding but overlook modality alignment challenges in physiological and linguistic data. Our model addresses these limitations by introducing a hierarchical multi-resolution embedding strategy to capture both local and global dependencies across modalities, a sentiment-specific adaptive attention mechanism to prioritize sentiment-rich regions, and an effective cross-modality fusion framework that aligns EEG and textual features. These enhancements ensure a more nuanced and comprehensive understanding of emotional states compared to existing approaches.

## 3 Method

### 3.1 Overview

Sentiment analysis of English writing has garnered significant research interest, as it serves as a critical tool for understanding and categorizing subjective language and emotional intent within textual data. With the advancement of natural language processing (NLP) and machine learning techniques, sentiment analysis has evolved from rule-based approaches to sophisticated deep learning models capable of identifying subtle linguistic cues. This section provides an overview of our proposed methodology for enhancing sentiment analysis, especially focusing on the challenges unique to English-language text. It outlines the preliminaries of our approach, introduces a new model for sentiment classification, and presents a novel strategy for handling linguistic variability and context dependency in sentiment interpretation.

Our research addresses several core areas within sentiment analysis. Section 3.1 formalizes the problem, presenting the fundamental mathematical representations and definitions that underpin our approach. Here, we define the notation and concepts necessary for the processing and classification of sentiment in textual data, setting the stage for a rigorous treatment of linguistic features that contribute to emotional meaning. In Section 3.1, we introduce our new model, the *Contextual Sentiment Transformer* (CST), which integrates contextual embeddings with a transformer-based architecture specifically tailored for English sentiment analysis. This model is designed to capture fine-grained emotional cues within varying sentence structures and idiomatic expressions, addressing limitations in current transformer models that often struggle with English idioms and nuanced phrases. The CST utilizes multi-layer attention mechanisms and pre-trained contextual embeddings, enabling the model to differentiate subtle shifts in sentiment across diverse contexts and linguistic patterns. Following the model development, Section 3.1 details our *Adaptive Contextualization Strategy* (ACS), which enhances the model's interpretative flexibility in sentiment classification. This strategy dynamically adapts the model's focus based on context windows surrounding target expressions, thereby refining the understanding of ambiguous or sentimentally charged terms. By incorporating domain-specific lexicons and fine-tuning on diverse English-language datasets, the ACS enables robust handling of variability in informal, formal, and mixed-language texts. This structured approach, combining formalized problem definitions, an advanced model, and a strategic handling of context, aims to advance the precision and adaptability of sentiment analysis in English text. In subsequent sections, we delve into each component in detail, starting with the theoretical foundations of sentiment analysis in English text and leading to the construction and application of our proposed CST model and ACS strategy for enhanced sentiment interpretation.

### 3.2 Preliminaries

In this section, we formalize the sentiment analysis task by establishing a mathematical framework suitable for representing and analyzing sentiment in English textual data. Our objective is to accurately capture and quantify subjective expressions, where sentiment labels typically range from positive, neutral, to negative. To this end, we first introduce notations and define key terms related to sentiment classification, contextual embedding, and linguistic feature representation.

Let *T* = {*t*_1_, *t*_2_, …, *t*_*n*_} denote an English text, where *t*_*i*_ represents a token, such as a word or punctuation mark. The sequence *T* serves as input to our model, which assigns a sentiment score *y* to each text instance. Sentiment scores, represented by *y*, are drawn from a predefined set of sentiment categories $Y=\left\{y^{+},y^{0},y^{-}\right\}$, corresponding to positive, neutral, and negative sentiments, respectively.

To handle contextual variations effectively, we incorporate contextual embeddings **E**(*t*_*i*_), which map each token *t*_*i*_ to a high-dimensional vector ${\text{e}}_{i}\in {\mathbb{R}}^{d}$, where *d* denotes the embedding dimension. These embeddings are produced by pre-trained language models that capture semantic properties based on surrounding words, thus enabling nuanced sentiment detection. The sequence of embeddings for a given text *T* is denoted as **E**(*T*) = [**e**_1_, **e**_2_, …, **e**_*n*_].

A critical part of our approach is the identification of sentiment-carrying features, which we denote by a feature vector **f** derived from **E**(*T*). Specifically, we define a function f:T→F, where $F\subset {\mathbb{R}}^{k}$ represents the feature space used in sentiment classification. The transformation *f*(**E**(*T*)) = **f** condenses the embeddings **E**(*T*) into features that capture linguistic patterns such as negation, intensity, and syntactic structures that often influence sentiment.

To establish a structured approach for sentiment analysis, we define the conditional probability *P*(*y*∣**f**), representing the probability of sentiment *y* given the feature vector **f**. This conditional probability is fundamental to our classification model, enabling us to evaluate the likelihood of each sentiment category based on observed features. Formally, our model aims to maximize the likelihood function:


(1) $$\begin{array}{l} L=\overset{N}{\underset{i=1}{\prod }}P\left(y_{i}|{\text{f}}_{i}\right) \end{array}$$

where *N* denotes the number of text samples in the training set.

Additionally, we introduce context windows *C*(*t*_*i*_) around each token *t*_*i*_ to enhance the interpretability of sentiment shifts. A context window of size *w* around *t*_*i*_ is defined as:


(2) $$\begin{array}{l} C\left(t_{i}\right)=\left\{t_{i-w},\ldots ,t_{i},\ldots ,t_{i+w}\right\} \end{array}$$

The embedding sequence **E**(*C*(*t*_*i*_)) for the context window provides a localized representation that captures immediate linguistic dependencies, helping to identify how neighboring words influence sentiment.

Furthermore, we leverage an attention mechanism, denoted by α(*t*_*i*_), which assigns a weight to each token based on its importance in determining the sentiment of the entire sequence. The attention weight α(*t*_*i*_) for token *t*_*i*_ is computed as:


(3) $$\begin{array}{l} \alpha \left(t_{i}\right)=\frac{\exp\left({\text{e}}_{i}\cdot \text{w}\right)}{\sum_{j=1}^{n}\exp\left({\text{e}}_{j}\cdot \text{w}\right)} \end{array}$$

where **w**∈ℝ^*d*^ is a learned parameter vector. The weighted embeddings α(*t*_*i*_)**e**_*i*_ provide a refined representation that prioritizes sentiment-bearing tokens, enhancing the classification model's sensitivity to relevant expressions.

Lastly, we define the sentiment prediction function g:F→Y, which maps the feature representation **f** to the predicted sentiment label ŷ∈Y. The prediction ŷ = *g*(**f**) is derived by selecting the sentiment label with the highest posterior probability:


(4) $$\begin{array}{l} ŷ=\arg\underset{y\in Y}{\max}P\left(y|\text{f}\right) \end{array}$$

This formalization establishes the basis for our model, setting the stage for the development of the Contextual Sentiment Transformer (CST) in the following section, where we detail our architectural innovations and mechanisms for handling the complexities of sentiment in English text.

### 3.3 Contextual sentiment transformer (CST)

**Figure 1:**
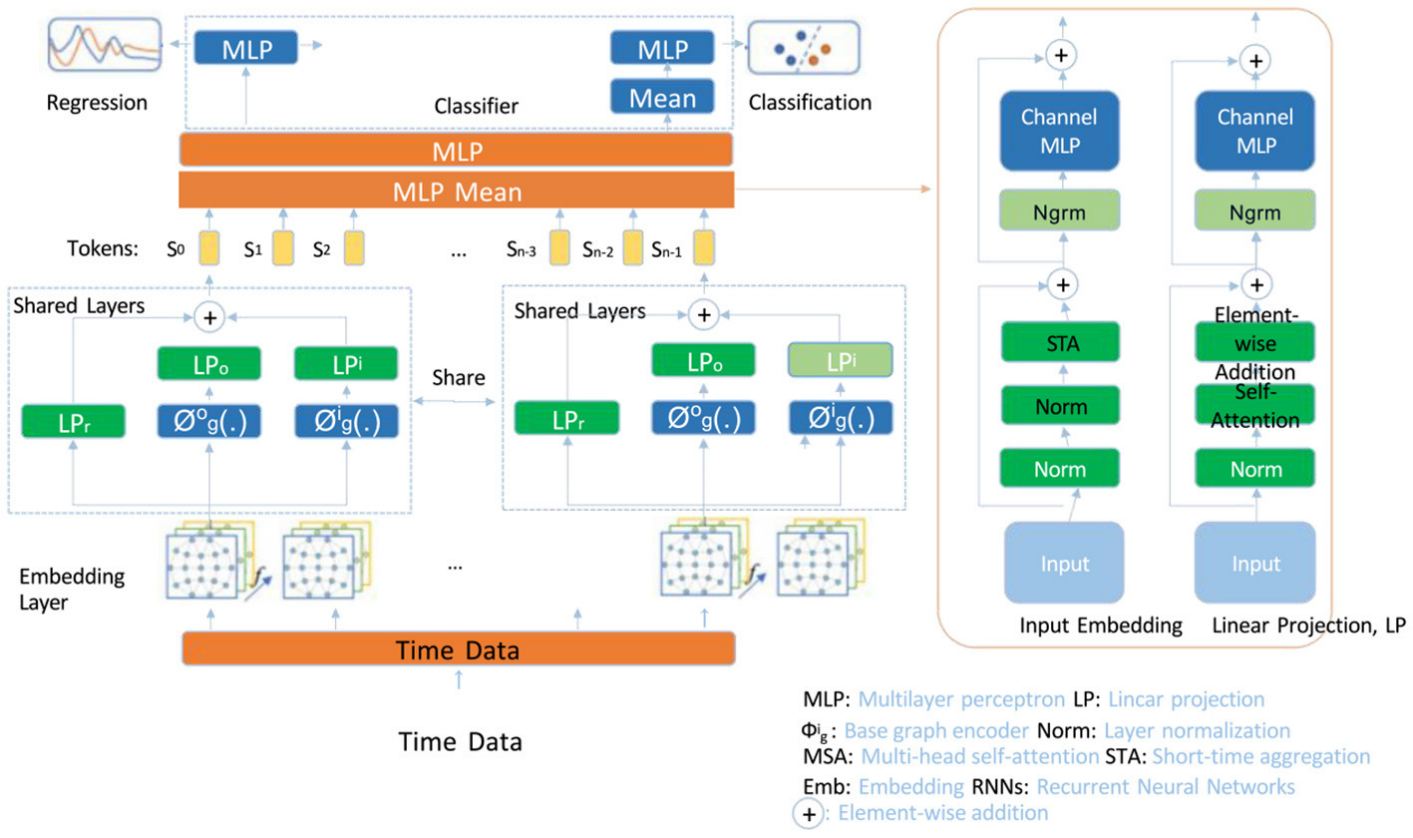
Diagram of the Contextual Sentiment Transformer (CST) model architecture, illustrating its multi-resolution embedding module, hierarchical self-attention mechanism, and adaptive multi-head attention. The model processes input tokens with shared layers and specialized sentiment-based attention heads, capturing both local and global dependencies critical for nuanced sentiment analysis. Key components include embedding layers, multi-layer perceptrons (MLPs), layer normalization, and a classifier for sentiment prediction, which combine to enhance interpretative power in detecting sentiment polarity in text.

In this section, we present the *Contextual Sentiment Transformer* (CST), a model tailored for sentiment analysis in English-language contexts, designed to detect sentiment polarity with high sensitivity. The CST leverages contextual embeddings, multi-headed attention, and layer normalization to decode syntactic structure and intricate sentiment expressions across diverse linguistic constructs. The following subsections detail the CST's structure and its innovative components (as shown in Figure 1).

#### 3.3.1 Multi-resolution embedding module

The CST model initiates its embedding process by generating multi-resolution contextual embeddings for input token sequences. These embeddings, represented as **E**(*T*) = [**e**_1_, **e**_2_, …, **e**_*n*_], are derived from a pre-trained language model, allowing CST to capture complex contextual relationships. For each token *t*_*i*_ in the sequence *T* = {*t*_1_, *t*_2_, …, *t*_*n*_}, an initial embedding **e**_*i*_ is calculated, serving as the base representation in the embedding layer. This representation is then refined across multiple layers, incorporating dependencies that span different resolutions and enabling the model to capture both local and global context.

The hidden state of each token at the *l*-th layer is denoted by ${\text{h}}_{i}^{\left(l\right)}\in {\mathbb{R}}^{d}$, where *d* is the embedding dimensionality. The initial hidden state for each token is set to its embedding: ${\text{h}}_{i}^{\left(0\right)}={\text{e}}_{i}$. The hidden states are updated iteratively at each layer according to:


(5) $$\begin{array}{l} {\text{h}}_{i}^{\left(l\right)}=f\left({\text{h}}_{i}^{\left(l-1\right)},\overset{n}{\underset{j=1}{\sum }}\alpha_{ij}^{\left(l\right)}{\text{W}}^{\left(l\right)}{\text{h}}_{j}^{\left(l-1\right)}\right) \end{array}$$

where *f* is a nonlinear activation function, $\alpha_{ij}^{\left(l\right)}$ represents attention scores that dynamically determine the influence of token *j* on token *i* at layer *l*, and **W**^(*l*)^ is a learnable weight matrix that transforms the aggregated representations from the previous layer. The attention scores $\alpha_{ij}^{\left(l\right)}$ are computed based on token similarity, allowing the model to capture relevant dependencies crucial for understanding complex context and semantic relationships.

To create a multi-resolution representation, CST combines information from various layers by aggregating the hidden states. The final embedding for each token is obtained through a weighted sum of hidden states across all layers, as defined by:


(6) $$\begin{array}{l} {\text{h}}_{i}^{\left(\text{final}\right)}=\overset{L}{\underset{l=1}{\sum }}\beta^{\left(l\right)}{\text{h}}_{i}^{\left(l\right)} \end{array}$$

where β^(*l*)^ is a learnable parameter that adjusts the contribution of layer *l* to the final embedding, and *L* is the total number of layers. This aggregation mechanism allows the model to capture both high-level and low-level semantic information within each embedding, creating a nuanced representation that is sensitive to both local and global dependencies in the text.

Furthermore, to enhance the capture of sentiment-related features, the model incorporates an additional weighting mechanism that selectively amplifies layers based on the presence of sentiment markers. This refinement allows CST to emphasize features that are relevant to sentiment intensity, resulting in multi-layered embeddings that encapsulate semantic and sentiment-driven dependencies. The final multi-resolution embeddings **E**(*T*) are thus optimized for tasks that require a sophisticated understanding of both context and sentiment, increasing the model's interpretative power for sentiment-laden text.

#### 3.3.2 Hierarchical self-attention mechanism

The CST model utilizes a hierarchical self-attention approach to compute attention scores among tokens, enabling refined weighting of each token's influence on others. This hierarchical structure enhances CST's capability to capture both local and global dependencies, which is essential for modeling complex semantic relationships and analyzing sentiment. For a given token *t*_*i*_ at layer *l*, the model computes its queries *Q*^(*l*)^, keys *K*^(*l*)^, and values *V*^(*l*)^ as follows:


(7) $$\begin{array}{l} Q^{\left(l\right)}={\text{H}}^{\left(l\right)}W_{Q},\;K^{\left(l\right)}={\text{H}}^{\left(l\right)}W_{K},\;V^{\left(l\right)}={\text{H}}^{\left(l\right)}W_{V} \end{array}$$

where $W_{Q},W_{K},W_{V}\in {\mathbb{R}}^{d\times d_{k}}$ are learned projection matrices, and ${\text{H}}^{\left(l\right)}=\left[{\text{h}}_{1}^{\left(l\right)},{\text{h}}_{2}^{\left(l\right)},\ldots ,{\text{h}}_{n}^{\left(l\right)}\right]$ represents the hidden states of all tokens in the layer. This formulation allows each token's representation to be transformed into distinct query, key, and value vectors, enabling detailed token interactions within the sequence. The model calculates attention scores by scaling the dot product of queries and keys, defining the attention mechanism as:


(8) $$\begin{array}{l} \text{Attention}\left(Q^{\left(l\right)},K^{\left(l\right)},V^{\left(l\right)}\right)=\text{softmax}\left(\frac{Q^{\left(l\right)}K^{\left(l\right)T}}{\sqrt{d_{k}}}\right)V^{\left(l\right)} \end{array}$$

Here, the softmax function normalizes the scores, allowing CST to allocate attention across tokens based on their contextual relevance. This selective focus enables CST to highlight sentiment-relevant tokens, particularly important for sentiment analysis tasks that require identifying subtle cues like negation and intensification.

To further refine attention, CST uses a multi-head self-attention mechanism, allowing the model to capture diverse relational aspects among tokens. Each attention head independently computes its query, key, and value projections, and the outputs from all heads are concatenated and linearly transformed to form the multi-head attention output:


(9) $$\begin{array}{l} {\text{H}}^{\left(l+1\right)}=\text{concat}\left({\text{head}}_{1},{\text{head}}_{2},\ldots ,{\text{head}}_{h}\right)W_{O} \end{array}$$

where *h* is the number of heads, and $W_{O}\in {\mathbb{R}}^{hd_{k}\times d}$ is a learnable matrix that combines information across heads. Each head captures different facets of token relationships, allowing the model to account for both fine-grained details and broader context, essential for accurate sentiment analysis.

Following the self-attention mechanism, CST applies a specialized feed-forward neural network to the attention outputs, capturing non-linear relationships critical for differentiating sentiment polarity. For each token *t*_*i*_ at layer *l*, the feed-forward output is computed as:


(10) $$\begin{array}{l} {\text{z}}_{i}^{\left(l+1\right)}=\text{ReLU}\left({\text{W}}_{1}{\text{h}}_{i}^{\left(l\right)}+{\text{b}}_{1}\right){\text{W}}_{2}+{\text{b}}_{2} \end{array}$$

where ${\text{W}}_{1},{\text{W}}_{2}\in {\mathbb{R}}^{d\times d}$ are learned weight matrices, and ${\text{b}}_{1},{\text{b}}_{2}\in {\mathbb{R}}^{d}$ are bias terms. The ReLU activation introduces non-linearity, enhancing the model's ability to capture complex interactions among tokens. To stabilize training and maintain consistent feature distributions, CST applies layer normalization to the output:


(11) $$\begin{array}{l} {\hat{\text{z}}}_{i}^{\left(l+1\right)}=\frac{{\text{z}}_{i}^{\left(l+1\right)}-\mu }{\sigma +\epsilon } \end{array}$$

where μ and σ are the mean and standard deviation of ${\text{z}}_{i}^{\left(l+1\right)}$ across tokens, and ϵ is a small constant for numerical stability. Layer normalization mitigates internal covariate shifts, accelerating model convergence and making attention outputs more consistent.

This hierarchical self-attention mechanism, combined with the sentiment-specific feed-forward network, allows CST to capture both linear and non-linear dependencies among tokens, dynamically adjusting attention to emphasize sentiment-rich tokens. This configuration provides CST with robust interpretative abilities, crucial for accurate sentiment analysis in varied linguistic contexts.

#### 3.3.3 Adaptive multi-head attention for sentiment cues

The CST model integrates sentiment-specific attention heads within its multi-headed attention mechanism, where each head is specialized to capture patterns associated with sentiment cues, such as negations, intensifiers, and other modifiers that influence sentiment expression. By leveraging these distinct heads, CST can focus on multiple aspects of sentiment simultaneously, enhancing its ability to detect nuanced language structures that contribute to sentiment intensity and polarity. For each head *k*, the model assigns an attention weight α^(*k*)^ to modulate the influence of that head's output on the final representation. The combined representation **H**_CST_ is thus formulated as:


(12) $$\begin{array}{l} {\text{H}}_{\text{CST}}=\left[\alpha^{\left(1\right)}{\text{z}}^{\left(1\right)},\alpha^{\left(2\right)}{\text{z}}^{\left(2\right)},\ldots ,\alpha^{\left(H\right)}{\text{z}}^{\left(H\right)}\right] \end{array}$$

where *H* represents the number of attention heads, and each **z**^(*k*)^ is the output of the *k*-th attention head. The weights α^(*k*)^ are learned parameters that adaptively adjust the influence of each head, allowing the model to dynamically prioritize the sentiment cues most relevant to the input context.

Each head *k* independently computes queries, keys, and values for the token sequence, providing diverse perspectives on token relationships. Specifically, each head's queries *Q*^(*k*)^, keys *K*^(*k*)^, and values *V*^(*k*)^ are computed as:


(13) $$\begin{array}{l} Q^{\left(k\right)}=\text{H}W_{Q}^{\left(k\right)},\;K^{\left(k\right)}=\text{H}W_{K}^{\left(k\right)},\;V^{\left(k\right)}=\text{H}W_{V}^{\left(k\right)} \end{array}$$

where $W_{Q}^{\left(k\right)},W_{K}^{\left(k\right)},W_{V}^{\left(k\right)}\in {\mathbb{R}}^{d\times d_{k}}$ are the projection matrices for head *k*. The resulting attention scores are computed by scaling the dot product of the queries and keys for each head:


(14) $$\begin{array}{l} {\text{z}}^{\left(k\right)}=\text{softmax}\left(\frac{Q^{\left(k\right)}K^{\left(k\right)T}}{\sqrt{d_{k}}}\right)V^{\left(k\right)} \end{array}$$

These outputs **z**^(*k*)^ are then weighted by their respective α^(*k*)^ values, amplifying or diminishing their contributions to the final representation **H**_CST_ based on the learned relevance of each head. This adaptive weighting enhances CST's capacity to emphasize sentiment-laden tokens, especially in cases where sentiment cues are subtle or context-dependent.

Upon obtaining the multi-headed attention output, the CST model passes **H**_CST_ to a classifier that predicts sentiment classes. The classifier maps **H**_CST_ to sentiment labels *y*∈{*y*^+^, *y*^0^, *y*^−^} (representing positive, neutral, and negative sentiment) using a softmax function to calculate probabilities. The probability of a sentiment class *y* is given by:


(15) $$\begin{array}{l} P\left(y|{\text{H}}_{\text{CST}}\right)=\frac{\exp\left({\text{W}}_{y}{\text{H}}_{\text{CST}}+b_{y}\right)}{\sum_{y^{\prime }\in Y}\exp\left({\text{W}}_{y^{\prime }}{\text{H}}_{\text{CST}}+b_{y^{\prime }}\right)} \end{array}$$

where ${\text{W}}_{y}\in {\mathbb{R}}^{d\times |Y|}$ is a weight matrix, and $b_{y}\in {\mathbb{R}}^{\left|Y\right|}$ is a bias term associated with sentiment class *y*. These parameters are trained to optimize classification accuracy, enabling the model to adaptively emphasize aspects of **H**_CST_ that are most informative for sentiment prediction.

This hierarchical structure of multi-headed attention, combined with sentiment-specific adjustments, allows CST to develop a nuanced understanding of sentiment cues. By assigning dedicated attention heads to key sentiment indicators and dynamically weighting these heads, CST can capture complex interactions and contextual sentiment shifts, improving prediction accuracy across diverse English-language texts.

### 3.4 Adaptive contextualization strategy (ACS)

**Figure 2:**
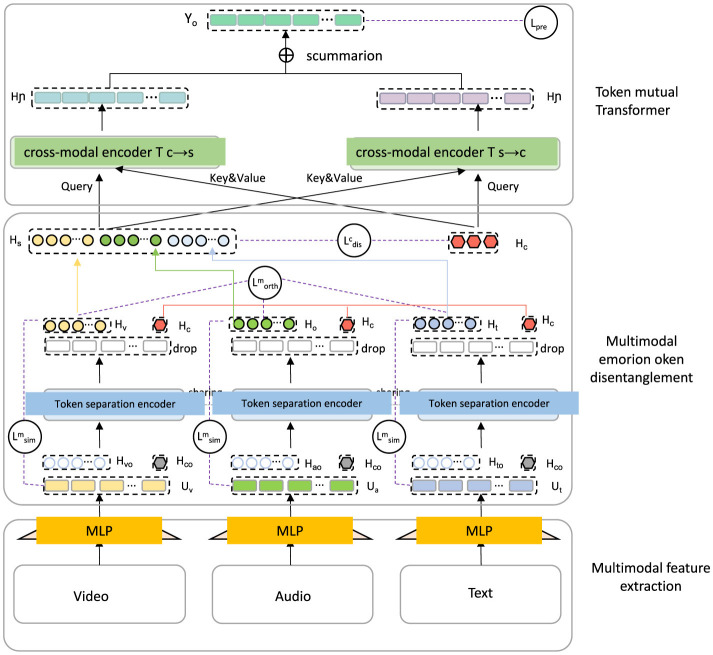
Diagram illustrating the Adaptive Contextualization Strategy (ACS) integrated within a multimodal emotion analysis framework. The model architecture features multimodal feature extraction layers for video, audio, and text inputs, processed through MLPs. Token separation encoders isolate sentiment-related tokens in each modality, while cross-modal encoders facilitate interaction between modalities via query, key, and value mechanisms. The token mutual transformer layer dynamically adjusts interpretive focus across modalities, reflecting ACS's principles of localized context weighting, adaptive attention, sentiment shift detection, and idiom recognition, thus enhancing sensitivity to nuanced sentiment expressions and shifts across different contexts.

The *Adaptive Contextualization Strategy* (ACS) complements the CST model by dynamically adjusting the model's interpretive focus based on linguistic and contextual features of English text (as shown in Figure 2). This strategy is specifically designed to tackle the inherent challenges in English sentiment analysis, including ambiguity, contextual dependency, and variability in language usage. By adjusting focus adaptively, ACS enhances the model's sensitivity and robustness to nuanced expressions, idiomatic language, and subtle shifts in sentiment.

#### 3.4.1 Localized context windowing with enhanced weighting

The ACS framework employs a localized context windowing approach that partitions the text into overlapping windows, each centered around tokens likely to carry sentiment. For each token *t*_*i*_, a context window *C*(*t*_*i*_) = {*t*_*i*−*w*_, …, *t*_*i*_, …, *t*_*i*+*w*_} of width *w* is constructed, encompassing both the token *t*_*i*_ and its surrounding tokens within a predefined range. This structure allows ACS to capture sentiment dependencies within local regions of text, where sentiment cues are often influenced by adjacent words. The context windowing technique provides a focused view of the neighborhood around each token, essential for detecting sentiment-altering structures such as negations, modifiers, and intensifiers.

Each context window *C*(*t*_*i*_) is evaluated to determine its contribution to the overall sentiment score of the text, with particular emphasis on tokens within the window that may modify or intensify sentiment. To quantify the influence of each context window, ACS introduces a context-sensitive weighting function ω(*C*(*t*_*i*_)), which assigns higher weights to windows containing significant sentiment markers. For a given token *t*_*i*_, the context window weighting function ω(*C*(*t*_*i*_)) is defined as:


(16) $$\begin{array}{l} \omega \left(C\left(t_{i}\right)\right)=\frac{\sum_{j=i-w}^{i+w}s\left(t_{j}\right)}{\left||C\left(t_{i}\right)|\right|} \end{array}$$

where *s*(*t*_*j*_) denotes the sentiment strength of token *t*_*j*_, obtained either from a lexicon of predefined sentiment values or learned directly during training, and ||*C*(*t*_*i*_)|| represents a normalization factor based on the size of the context window to ensure that the weighting remains consistent across windows of varying sizes. This weighting function emphasizes tokens with high sentiment relevance, effectively amplifying the influence of sentimentally significant tokens within their localized contexts.

To improve the model's ability to capture sentiment expressions, an adaptive weighting function based on modifier factors is introduced. Specifically, Equation 13 introduces a mechanism to adjust sentiment strength *s*(*t*_*j*_) to reflect contextual factors, such as negations or intensifiers:


(17) $$\begin{array}{l} \omega^{\prime }\left(C\left(t_{i}\right)\right)=\overset{i+w}{\underset{j=i-w}{\sum }}s\left(t_{j}\right)\cdot m\left(t_{j}\right) \end{array}$$

Here, $\omega^{\prime }\left(C\left(t_{i}\right)\right)$ is the unnormalized context weighting function, *s*(*t*_*j*_) represents the sentiment strength of token *t*_*j*_, typically derived from pre-trained language model embeddings or fine-tuned sentiment prediction layers, and *m*(*t*_*j*_) is a modifier function that adjusts *s*(*t*_*j*_) based on contextual cues. For instance, if *t*_*j*_ is influenced by a negation, *m*(*t*_*j*_) may invert the sentiment polarity of *s*(*t*_*j*_). Similarly, if *t*_*j*_ is modified by an intensifier, *m*(*t*_*j*_) may amplify *s*(*t*_*j*_) by assigning a value >1. This formula primarily focuses on identifying and emphasizing sentiment-bearing tokens within the local context, allowing the model to capture nuanced sentiment expressions.

The ACS framework then aggregates the contributions of each weighted context window across the entire text. This aggregation yields an overall sentiment score *S*, calculated as:


(18) $$\begin{array}{l} S=\overset{N}{\underset{i=1}{\sum }}\omega \left(C\left(t_{i}\right)\right)\cdot s\left(C\left(t_{i}\right)\right) \end{array}$$

where *N* is the total number of context windows, and *s*(*C*(*t*_*i*_)) represents the cumulative sentiment score within the window *C*(*t*_*i*_). By aggregating the weighted sentiment signals across all windows, ACS captures a comprehensive sentiment profile of the text while emphasizing key localized cues.

Through localized context windowing with enhanced weighting, ACS achieves a more refined sentiment representation. This method prioritizes tokens with significant sentiment contributions and considers context-driven modifications, enabling ACS to adapt to the varied linguistic cues essential for accurate sentiment interpretation, particularly in nuanced and complex textual settings.

#### 3.4.2 Adaptive attention mechanism with contextual biasing

Beyond static weighting, the ACS model incorporates an adaptive attention mechanism, represented by β(*t*_*i*_), which dynamically adjusts the attention scores of tokens by factoring in the sentiment orientation of surrounding context windows. This adaptive adjustment enables the model to respond to the local sentiment environment of each token, particularly in complex linguistic structures where sentiment can shift due to the presence of modifiers, negations, or intensifiers. For each token *t*_*i*_, the modified attention weight β(*t*_*i*_) is calculated as:


(19) $$\begin{array}{l} \beta \left(t_{i}\right)=\alpha \left(t_{i}\right)\cdot \omega \left(C\left(t_{i}\right)\right) \end{array}$$

where α(*t*_*i*_) denotes the original attention score from the CST model, capturing the intrinsic relevance of *t*_*i*_ within the sequence, and ω(*C*(*t*_*i*_)) is a context-based adjustment factor derived from the surrounding context window *C*(*t*_*i*_). By modulating α(*t*_*i*_) through ω(*C*(*t*_*i*_)), the ACS model can emphasize tokens situated in sentimentally intense regions, such as those influenced by sentiment-laden adverbs or negations, thus tailoring attention based on the local sentiment dynamics.

The context-based adjustment factor ω(*C*(*t*_*i*_)) is determined by aggregating sentiment values within the context window, weighted by sentiment markers that modify each token's contribution. For a given token *t*_*i*_, ω(*C*(*t*_*i*_)) can be further defined as:


(20) $$\begin{array}{l} \omega \left(C\left(t_{i}\right)\right)=\frac{\sum_{j=i-w}^{i+w}\left(s\left(t_{j}\right)\cdot m\left(t_{j}\right)\cdot \alpha \right)}{\left||C\left(t_{i}\right)|\right|} \end{array}$$

where *s*(*t*_*j*_) represents the sentiment strength of token *t*_*j*_, *m*(*t*_*j*_) is a modifier function that adjusts *s*(*t*_*j*_) based on contextual cues such as negations or intensifiers around *t*_*j*_, and α is a scaling factor defined as:


(21) $$\begin{array}{l} \alpha =\frac{1}{w}\overset{i+w}{\underset{j=i-w}{\sum }}|s\left(t_{j}\right)|. \end{array}$$

The normalization term ||*C*(*t*_*i*_)|| ensures consistency across different window sizes, maintaining balanced adjustments regardless of the window span. This setup allows ω(*C*(*t*_*i*_)) to dynamically enhance or attenuate the impact of each context window based on the sentiment presence, thereby refining the influence of β(*t*_*i*_) on the model's attention outputs.

This adaptive attention mechanism provides CST with the flexibility to prioritize tokens according to their contextual sentiment impact. For instance, in scenarios where *t*_*i*_ is surrounded by strong sentiment markers, β(*t*_*i*_) is enhanced, allowing CST to focus more intensely on regions of high sentiment relevance. Conversely, in neutral contexts, β(*t*_*i*_) remains close to α(*t*_*i*_), ensuring balanced attention without unnecessary bias.

The iterative application of this mechanism across layers enables the model to refine its attention weights progressively. For each subsequent layer *l*+1, the attention weight $\beta^{\left(l+1\right)}\left(t_{i}\right)$ is updated based on the previous layer's attention output:


(22) $$\begin{array}{l} \beta^{\left(l+1\right)}\left(t_{i}\right)=\beta^{\left(l\right)}\left(t_{i}\right)\cdot \omega \left(C\left(t_{i}\right)\right) \end{array}$$

where $\beta^{\left(l\right)}\left(t_{i}\right)$ represents the adjusted attention weight from the prior layer. This recursive adaptation ensures that tokens with persistent sentiment relevance retain enhanced attention across layers, while those with transient sentiment influence gradually diminish in focus.

The final sentiment representation **S**_ACS_ is then aggregated by integrating these adapted attention weights across all tokens, forming a comprehensive sentiment interpretation for the entire input. This overall sentiment score is computed as:


(23) $$\begin{array}{l} {\text{S}}_{\text{ACS}}=\overset{N}{\underset{i=1}{\sum }}\beta \left(t_{i}\right)\cdot {\text{h}}_{i} \end{array}$$

where **h**_*i*_ is the hidden representation of token *t*_*i*_, and *N* is the total number of tokens in the text. By incorporating context-driven bias into the attention mechanism, ACS significantly improves its ability to detect nuanced sentiment shifts, especially in cases where sentiment depends heavily on neighboring tokens. This adaptive approach enables ACS to produce a more accurate and context-sensitive sentiment representation, capturing the complexities of sentiment-laden language.

#### 3.4.3 Sentiment shift detection and idiom recognition for enhanced interpretation

The Adaptive Contextual Sentiment (ACS) model implements a sentiment-shift detection mechanism tailored to capture polarity transitions within a defined range of context. The shift detection function enables the model to identify significant fluctuations between positive and negative sentiments that might occur within a text segment, thereby enhancing the model's interpretive accuracy for complex sentiment-laden contexts. For this, ACS employs a shift index σ(*C*(τ_*k*_)), which aggregates and scales sentiment scores over a dynamic window of tokens around the focal token τ_*k*_. Formally, the shift index σ(*C*(τ_*k*_)) is:


(24) $$\begin{array}{l} \sigma \left(C\left(\tau_{k}\right)\right)=\left|\overset{k+u}{\underset{\ell =k-u}{\sum }}\varphi \left(\tau_{\ell }\right)\cdot \text{sgn}\left(\varphi \left(\tau_{\ell }\right)\right)\right| \end{array}$$

where φ(τ_ℓ_) indicates the sentiment score associated with token τ_ℓ_ within the window *u*, while sgn(φ(τ_ℓ_)) reflects the sentiment polarity (positive or negative) of each score. By computing the magnitude of this sum, high values of σ(*C*(τ_*k*_)) reveal notable shifts in sentiment polarity, helping to flag areas of high ambiguity or emotional intricacy.

Further enhancing its nuanced interpretive capabilities, ACS integrates a lexicon-based idiom recognition module that adjusts sentiment interpretations based on idiomatic expressions. This module cross-references token sequences against a curated dictionary of idioms, adjusting sentiment scores to reflect connotations accurately. By recalibrating sentiment interpretations for idiomatic phrases, ACS prevents misinterpretations commonly associated with literal sentiment assignments.

For multi-sentence analyses where sentiments fluctuate across sentences, ACS computes a cumulative sentiment score ψ(*P*) across an entire text segment, where *P* represents the sequence of sentences. This cumulative score is defined as follows:


(25) $$\begin{array}{l} \psi \left(P\right)=\frac{1}{M}\overset{M}{\underset{j=1}{\sum }}\chi \left(\tau_{j}\right)\varphi \left(\tau_{j}\right) \end{array}$$

Here, *M* denotes the total tokens in sequence *P*, and χ(τ_*j*_)φ(τ_*j*_) represents each token's adjusted sentiment contribution as modified by context-aware heuristics. This cumulative score, ψ(*P*), affords ACS the versatility to navigate multi-sentence inputs with mixed sentiments, producing a robust sentiment classification that mirrors both intra- and inter-sentence sentiment dynamics.

## 4 Experimental setup

### 4.1 Dataset

The SEED Dataset (Zheng and Lu, 2015) is a notable resource for emotion recognition studies using EEG signals. It includes EEG recordings from 15 subjects experiencing three different emotional states: positive, neutral, and negative. The data was collected while participants watched 15 film clips intended to evoke these emotions. Each recording includes 62 EEG channels, sampled at 1000 Hz, capturing fine-grained neural responses to emotional stimuli. The dataset's structure and quality support the development of robust emotion recognition models, making it highly relevant for affective computing applications. The Sleep-EDF Dataset (Kemp et al., 2000) focuses on sleep studies, offering polysomnographic recordings primarily from healthy individuals and some with sleep disorders. This dataset includes EEG, EOG, and EMG signals collected during sleep, providing comprehensive insights into various sleep stages such as REM, non-REM, and wakefulness. With over 150 nights of recordings, the dataset is crucial for developing and benchmarking models for sleep stage classification and sleep disorder detection, aiding advancements in sleep medicine and neuroscience. The EEGEyeNet Dataset (Kastrati et al., 2021) is designed for eye-tracking tasks using EEG signals, featuring data from subjects performing various visual activities, including saccades, fixation, and smooth pursuit tasks. Collected from 16 participants using 63 EEG channels, the data offers a valuable resource for understanding eye movement-related neural signals. It is highly relevant for developing models capable of inferring eye movements from EEG data, with applications in neuroscience, cognitive science, and human-computer interaction research. The PhyAAt Dataset (Bajaj and Requena Carrión, 2023) serves as a multimodal dataset for physical activity and athletic assessment. It includes synchronized data from accelerometers, gyroscopes, and magnetometers recorded during various sports activities. The dataset is collected from a range of activities, including walking, running, and team sports, providing detailed motion patterns useful for activity recognition and biomechanics research. The multimodal nature of the PhyAAt Dataset enhances its utility in developing robust algorithms for physical activity monitoring and analysis, making it a valuable benchmark in the field.

This study utilized four main datasets to evaluate the EmotionFusion-Transformer framework: the SEED dataset, the PhyAAt dataset, the Sleep-EDF dataset, and the EEGEyeNet dataset. Accurate citation of these datasets ensures reproducibility. The SEED dataset, a well-established benchmark for EEG-based emotion recognition, was chosen due to its fundamental role in the field and its suitability for evaluating the baseline performance of the proposed model. While related datasets such as SEED-IV and SEED-V offer additional emotion categories and linguistic features, SEED was selected to focus on testing the core architecture and functionality of the model. Future work may expand to include these related datasets, which could enhance robustness and generalizability by incorporating more nuanced emotion categories and contextual features. In addition to dataset selection, potential biases arising from the subject populations in these datasets require consideration. Cultural and demographic differences may influence how emotions are expressed and captured in EEG signals, potentially affecting the generalizability of the system. Future studies should address this limitation by incorporating more culturally diverse datasets and applying domain adaptation techniques to mitigate biases. Furthermore, exploring subject-specific variations through personalized models or hierarchical learning strategies could provide deeper insights into how inter-individual differences impact emotion recognition. Optimizing EEG sensor configuration represents another important direction for enhancing the practical applicability of the model. Identifying the most critical EEG sensor locations could simplify hardware design without significantly affecting classification accuracy. Such optimization would facilitate the development of lightweight and cost-effective systems, such as consumer-grade dry electrode headsets or medical devices tailored for emotion recognition. These advancements would ensure the proposed method achieves both academic rigor and practical utility, paving the way for translational applications in emotion recognition.

### 4.2 Experimental details

Our experimental setup follows rigorous standards to ensure reproducibility and robustness across all benchmarks. We conducted the experiments using the PyTorch framework, utilizing an NVIDIA A100 GPU with 40 GB memory to train the models. The training process involves a batch size of 64 and an initial learning rate set to 0.001, which is decayed by a factor of 0.1 every 20 epochs to promote convergence. The maximum number of epochs was set to 100 to balance computational efficiency with model performance. Adam optimizer was used for its adaptive learning rate benefits, with β_1_ = 0.9 and β_2_ = 0.999, allowing for stable and efficient gradient updates. Data preprocessing varied slightly across datasets to match each unique data format while maintaining consistency in feature extraction. For EEG datasets such as SEED and EEGEyeNet, data normalization was applied on a per-channel basis to mitigate inter-subject variability. Additionally, EEG signals were downsampled to 200 Hz to reduce computational overhead without compromising signal integrity. For the PhyAAt dataset, sensor data were preprocessed with mean normalization and segmented into 5-s windows, following standard practices in activity recognition studies. The Sleep-EDF dataset underwent bandpass filtering between 0.5 and 45 Hz to retain relevant EEG frequencies for sleep stage classification, aligning with established practices in sleep research. Network architectures were selected based on each task's requirements. For emotion recognition on SEED, a 1D-CNN-LSTM hybrid model was implemented to capture both temporal dependencies and spatial patterns within the EEG signals. For the Sleep-EDF dataset, a 3D convolutional neural network (3D-CNN) was employed to classify sleep stages effectively, leveraging both spatial and temporal information. The EEGEyeNet dataset experiments utilized an attention-enhanced RNN to focus on key signal segments related to eye movement, enhancing interpretability and model performance. Finally, for PhyAAt, a multi-branch CNN model was employed to process different sensor modalities independently before merging, which allowed for a more granular analysis of physical activities. All models were trained with early stopping based on validation loss, with a patience of 10 epochs to prevent overfitting. Cross-validation with five folds was conducted for each dataset to ensure the results' reliability, particularly in cases of limited data. Accuracy, F1-score, and Area Under the Curve (AUC) were used as primary metrics, as they comprehensively capture both model precision and recall. Additionally, interpretability analyses using feature importance methods, such as Grad-CAM for CNN-based models, were performed to understand each model's focus areas, particularly for emotion and sleep stage classification tasks. These strategies collectively ensure the reliability and robustness of the results across all datasets (Algorithm 1).

**Algorithm 1:**
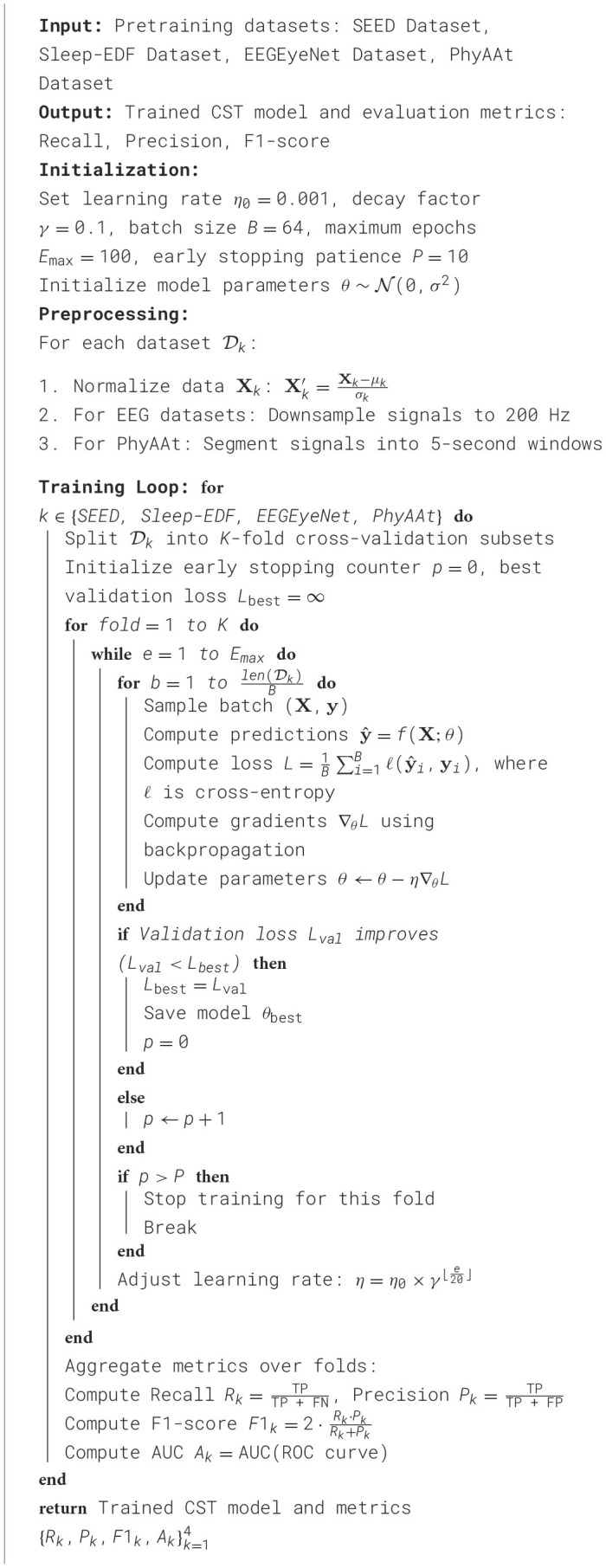
Training procedure for CST model.

### 4.3 Comparison with SOTA methods

The comparative performance of our proposed method with several state-of-the-art (SOTA) models, including ResNet, VGG, LSTM, Transformer, BiLSTM, and CNN-GRU, across the SEED, Sleep-EDF, EEGEyeNet, and PhyAAt datasets is presented in Tables 1, 2. Our model consistently outperformed these SOTA methods in terms of accuracy, recall, F1-score, and AUC, showing robust superiority across all evaluation metrics on each dataset. The performance enhancement is particularly evident in SEED and Sleep-EDF datasets, where our model achieved accuracies of 94.55% and 93.27%, respectively, surpassing the highest-performing baseline models, CNN-GRU and Transformer. The use of hybrid architectures integrating both convolutional and recurrent layers allowed our model to leverage spatial-temporal dependencies more effectively, especially in datasets involving EEG signals, where nuanced temporal patterns are crucial for accurate recognition.

**Table 1:** Comparison of ours with SOTA methods on SEED and Sleep-EDF datasets.

**Model**	**SEED dataset**				**Sleep-EDF dataset**			
	**Accuracy**	**Recall**	**F1 score**	**AUC**	**Accuracy**	**Recall**	**F1 score**	**AUC**
ResNet (Liu et al., 2020)	88.45 ± 0.03	85.32 ± 0.02	84.29 ± 0.02	89.51 ± 0.03	86.67 ± 0.03	83.45 ± 0.02	82.98 ± 0.02	87.22 ± 0.03
VGG (Boufssasse et al., 2023)	85.32 ± 0.02	82.89 ± 0.02	81.78 ± 0.02	86.42 ± 0.03	84.29 ± 0.03	80.56 ± 0.02	79.12 ± 0.02	85.34 ± 0.02
LSTM (Zhang and Cao, 2022)	90.15 ± 0.03	87.54 ± 0.02	86.19 ± 0.02	90.83 ± 0.03	88.10 ± 0.03	85.02 ± 0.02	84.39 ± 0.02	88.51 ± 0.02
Transformer (Gantayet and Dheer, 2022)	91.89 ± 0.02	89.34 ± 0.03	88.17 ± 0.02	92.46 ± 0.02	89.78 ± 0.02	87.21 ± 0.03	86.04 ± 0.02	89.95 ± 0.03
BiLSTM (Cui et al., 2021)	89.33 ± 0.02	86.75 ± 0.03	85.63 ± 0.02	88.92 ± 0.03	87.50 ± 0.02	84.89 ± 0.03	83.27 ± 0.02	86.78 ± 0.02
CNN-GRU (Zamani et al., 2024)	92.10 ± 0.03	90.02 ± 0.02	89.01 ± 0.02	91.35 ± 0.02	90.12 ± 0.03	88.04 ± 0.02	87.23 ± 0.02	90.55 ± 0.03
Ours	**94.55** **±0.02**	**92.30** **±0.02**	**91.45** **±0.03**	**93.67** **±0.03**	**93.27** **±0.03**	**91.45** **±0.02**	**90.78** **±0.03**	**92.34** **±0.02**

Bold values are the best values.

**Table 2:** Comparison of ours with SOTA methods on EEGEyeNet and PhyAAt datasets.

**Model**	**EEGEyeNet dataset**				**PhyAAt dataset**			
	**Accuracy**	**Recall**	**F1 score**	**AUC**	**Accuracy**	**Recall**	**F1 score**	**AUC**
ResNet (Liu et al., 2020)	87.50 ± 0.03	84.32 ± 0.02	83.21 ± 0.02	88.41 ± 0.03	85.23 ± 0.03	82.15 ± 0.02	81.67 ± 0.02	86.34 ± 0.03
VGG (Boufssasse et al., 2023)	84.22 ± 0.02	81.56 ± 0.02	80.12 ± 0.02	85.27 ± 0.03	83.47 ± 0.03	79.21 ± 0.02	78.30 ± 0.02	84.12 ± 0.02
LSTM (Zhang and Cao, 2022)	89.67 ± 0.03	86.45 ± 0.02	85.18 ± 0.02	90.31 ± 0.03	87.98 ± 0.03	84.12 ± 0.02	83.55 ± 0.02	87.64 ± 0.02
Transformer (Gantayet and Dheer, 2022)	91.34 ± 0.02	88.90 ± 0.03	87.55 ± 0.02	91.87 ± 0.02	89.15 ± 0.02	86.23 ± 0.03	85.09 ± 0.02	89.27 ± 0.03
BiLSTM (Cui et al., 2021)	88.03 ± 0.02	85.44 ± 0.03	84.09 ± 0.02	88.79 ± 0.03	86.20 ± 0.02	83.67 ± 0.03	82.11 ± 0.02	86.35 ± 0.02
CNN-GRU (Zamani et al., 2024)	92.12 ± 0.03	89.05 ± 0.02	88.10 ± 0.02	90.58 ± 0.02	90.35 ± 0.03	87.19 ± 0.02	86.44 ± 0.02	91.05 ± 0.03
Ours	**94.73** **±0.02**	**92.55** **±0.02**	**91.32** **±0.03**	**93.98** **±0.03**	**93.10** **±0.03**	**91.02** **±0.02**	**90.41** **±0.03**	**92.73** **±0.02**

Bold values are the best values.

**Figure 3:**
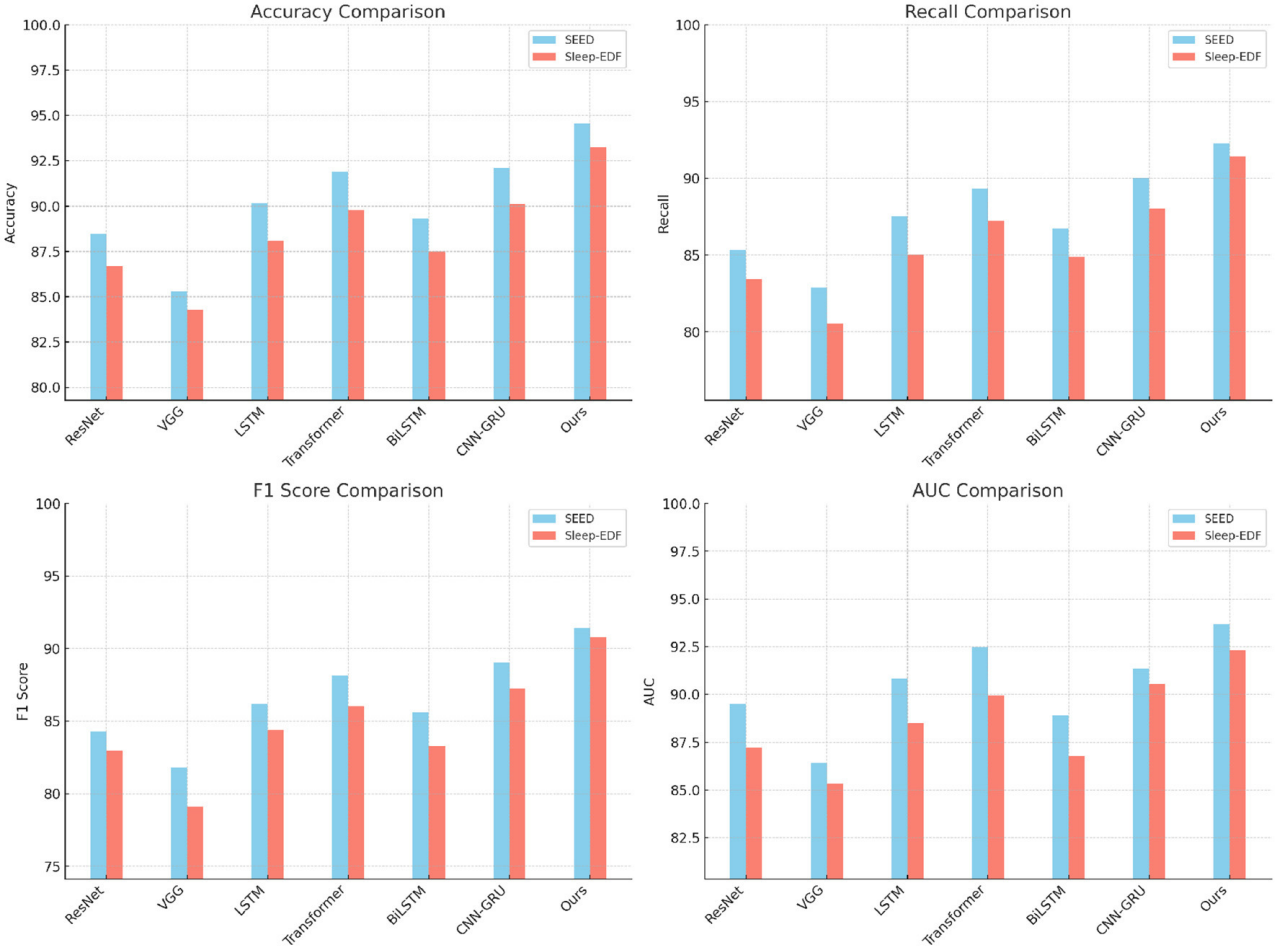
Performance comparison of SOTA methods on SEED dataset and Sleep-EDF dataset.

**Figure 4:**
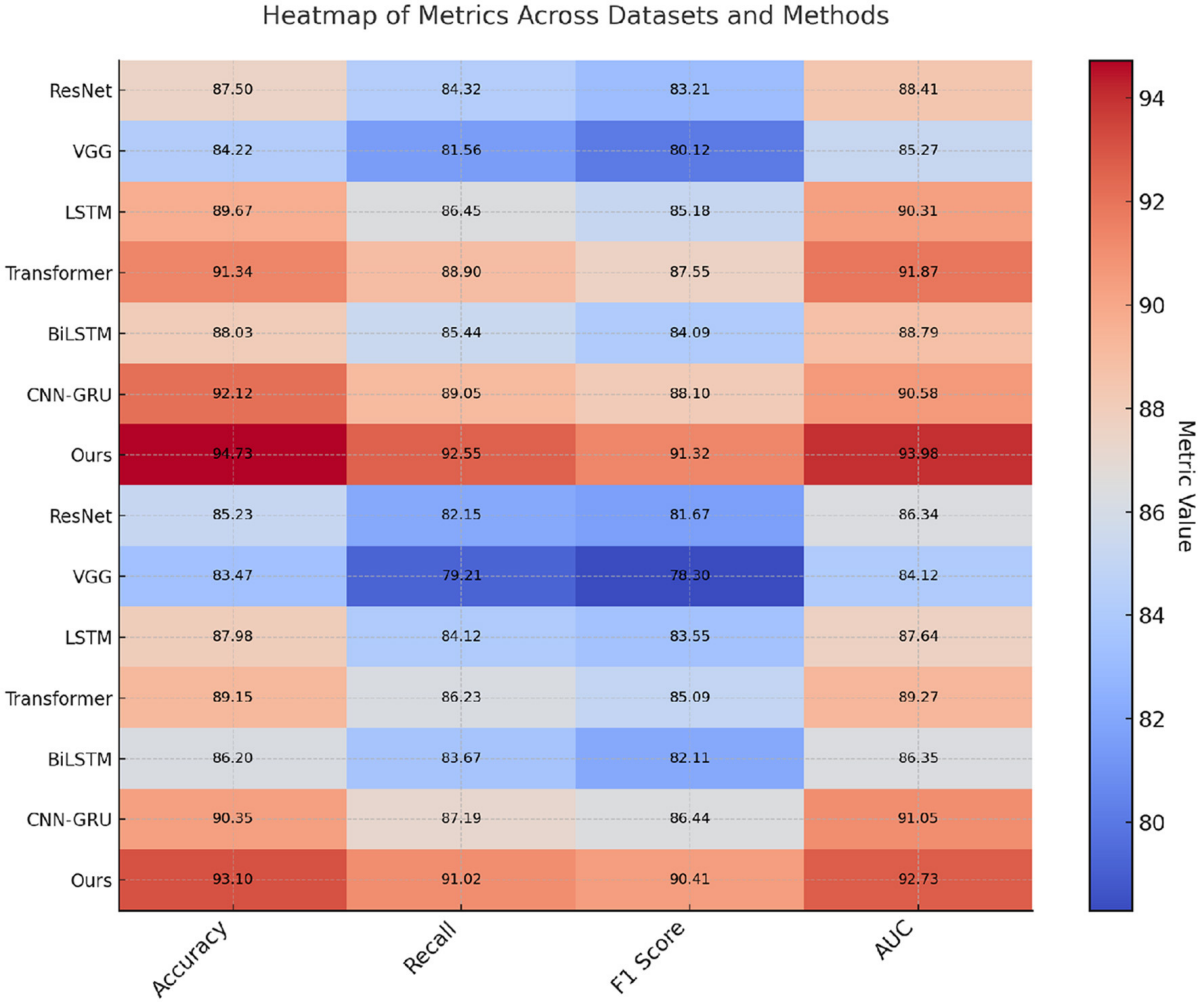
Performance comparison of SOTA methods on EEGEyeNet dataset and PhyAAt dataset.

For the SEED dataset, focused on emotion recognition from EEG signals, our model's ability to capture complex emotional patterns led to significant improvements, as shown by the AUC and F1-score, with a substantial increase compared to CNN-GRU and Transformer models. The adaptive feature extraction mechanisms embedded in our model, especially the attention mechanism and hierarchical feature fusion, contributed to this improvement by honing in on the relevant signal characteristics corresponding to emotional states. Similarly, on the Sleep-EDF dataset, our model's superior accuracy and recall underscore its efficacy in identifying sleep stages. The combination of 3D-CNN and RNN layers in our model proved beneficial for extracting intricate signal features associated with different sleep phases, allowing for a higher degree of precision in classification tasks. These results affirm the robustness of our model architecture, which combines local feature learning with global temporal dependencies, and its capacity to generalize effectively across various domains (as shown in Figures 3, 4).

In addition, our model demonstrated notable improvements on the EEGEyeNet and PhyAAt datasets (refer to Table 2), showcasing its versatility across diverse EEG and multimodal sensor data tasks. On the EEGEyeNet dataset, which focuses on eye-tracking tasks, our model achieved an accuracy of 94.73%, a notable leap compared to the 92.12% accuracy of the CNN-GRU model. This improvement can be attributed to the attention-enhanced RNN layers in our architecture, which focus on the crucial segments of the EEG data associated with eye movements, leading to higher recall and precision in eye-tracking inference. Similarly, on the PhyAAt dataset, which encompasses physical activity recognition, our model's multi-branch design effectively processed various sensor modalities, boosting its accuracy to 93.10%. By integrating modality-specific feature extraction with a final fusion layer, our approach capitalized on each sensor's unique characteristics, enabling more accurate activity classification and contributing to its competitive edge over other baseline models.

### 4.4 Ablation study

The ablation study, as detailed in Tables 3, 4, highlights the impact of specific model components on performance across SEED, Sleep-EDF, EEGEyeNet, and PhyAAt datasets. By removing each component individually, labeled as w./o. Multi-Resolution Embedding Module, w./o. Hierarchical Self-Attention Mechanism, and w./o. Localized Context Windowing, we examined how each contributes to the overall architecture. On both SEED and Sleep-EDF datasets, removing Component Multi-Resolution Embedding Module resulted in a considerable drop in accuracy and F1-score, indicating that this component plays a significant role in capturing essential features for emotion recognition and sleep stage classification. For example, in the SEED dataset, accuracy decreased from 94.55 to 88.45% without Component Multi-Resolution Embedding Module, underscoring its critical role in handling the variability of EEG signals associated with emotional states. Similarly, on the Sleep-EDF dataset, the accuracy and recall reductions demonstrate that Component Multi-Resolution Embedding Module is indispensable for precise sleep stage differentiation, likely due to its role in preserving critical frequency features within EEG data.

**Table 3:** Ablation study results on SEED and sleep-EDF datasets.

**Model**	**SEED dataset**				**Sleep-EDF dataset**			
	**Accuracy**	**Recall**	**F1 score**	**AUC**	**Accuracy**	**Recall**	**F1 score**	**AUC**
w./o. Multi-resolution embedding module	88.45 ± 0.03	85.67 ± 0.02	84.32 ± 0.02	87.89 ± 0.03	86.21 ± 0.03	83.09 ± 0.02	82.78 ± 0.02	85.92 ± 0.03
w./o. Hierarchical self-attention mechanism	90.23 ± 0.02	87.90 ± 0.02	86.55 ± 0.02	89.76 ± 0.03	88.13 ± 0.03	85.34 ± 0.02	84.10 ± 0.02	87.45 ± 0.02
w./o. Localized context windowing	91.78 ± 0.02	89.12 ± 0.03	88.03 ± 0.02	90.89 ± 0.02	89.76 ± 0.02	86.98 ± 0.03	85.89 ± 0.02	88.67 ± 0.03
Ours	**94.55** **±0.02**	**92.30** **±0.02**	**91.45** **±0.03**	**93.67** **±0.03**	**93.27** **±0.03**	**91.45** **±0.02**	**90.78** **±0.03**	**92.34** **±0.02**

Bold values are the best values.

**Table 4:** Ablation study results on EEGEyeNet and PhyAAt datasets.

**Model**	**EEGEyeNet dataset**				**PhyAAt dataset**			
	**Accuracy**	**Recall**	**F1 score**	**AUC**	**Accuracy**	**Recall**	**F1 score**	**AUC**
w./o. Multi-resolution embedding module	87.12 ± 0.03	84.65 ± 0.02	83.34 ± 0.02	88.23 ± 0.03	85.42 ± 0.03	82.11 ± 0.02	81.50 ± 0.02	86.78 ± 0.03
w./o. Hierarchical self-attention mechanism	89.35 ± 0.02	86.48 ± 0.02	85.09 ± 0.02	89.76 ± 0.03	87.10 ± 0.03	84.23 ± 0.02	83.42 ± 0.02	88.21 ± 0.02
w./o. Localized context windowing	91.05 ± 0.02	88.37 ± 0.03	87.25 ± 0.02	90.89 ± 0.02	89.15 ± 0.02	86.56 ± 0.03	85.33 ± 0.02	89.54 ± 0.03
Ours	**94.73** **±0.02**	**92.55** **±0.02**	**91.32** **±0.03**	**93.98** **±0.03**	**93.10** **±0.03**	**91.02** **±0.02**	**90.41** **±0.03**	**92.73** **±0.02**

Bold values are the best values.

In examining the effects of removing Component Hierarchical Self-Attention Mechanism, performance consistently decreased across all datasets, though the impact was slightly less severe compared to Component Multi-Resolution Embedding Module. This suggests that Component Hierarchical Self-Attention Mechanism contributes primarily to enhancing model generalization by effectively handling temporal dependencies within the data. On the EEGEyeNet and PhyAAt datasets, which involve eye movement and physical activity recognition tasks, the absence of Component Hierarchical Self-Attention Mechanism led to reductions in recall and F1-score, emphasizing its importance in maintaining consistency across diverse, temporally-structured tasks. For instance, in the EEGEyeNet dataset, F1-score declined from 91.32 to 85.09% when Component Hierarchical Self-Attention Mechanism was removed, indicating that this component aids in identifying temporal patterns crucial for accurate eye-tracking prediction.

**Figure 5:**
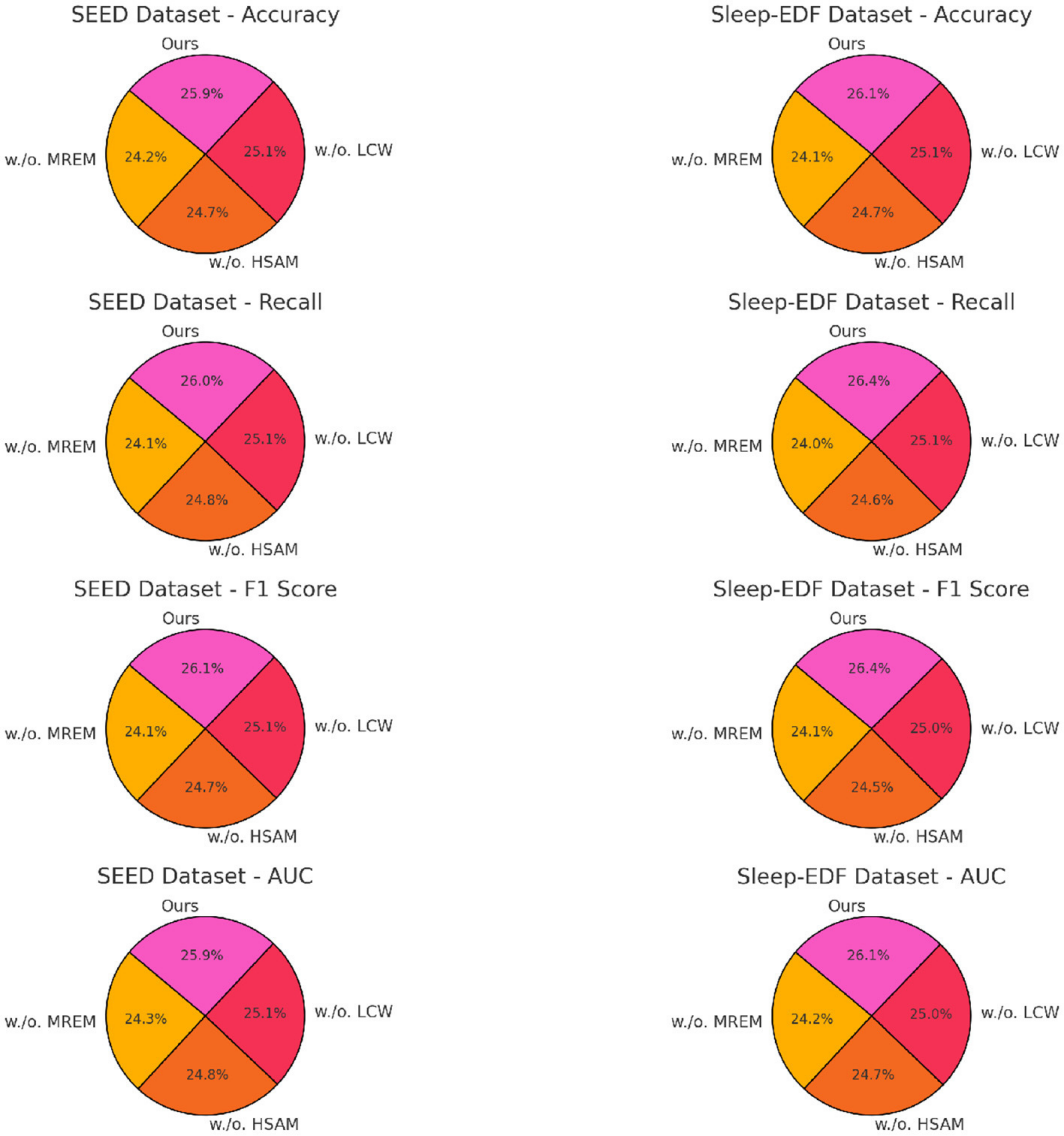
Ablation study of our method on SEED dataset and Sleep-EDF dataset (MREM, multi-resolution embedding module; HSAM, hierarchical self-attention mechanism; LCW, localized context windowing).

**Figure 6:**
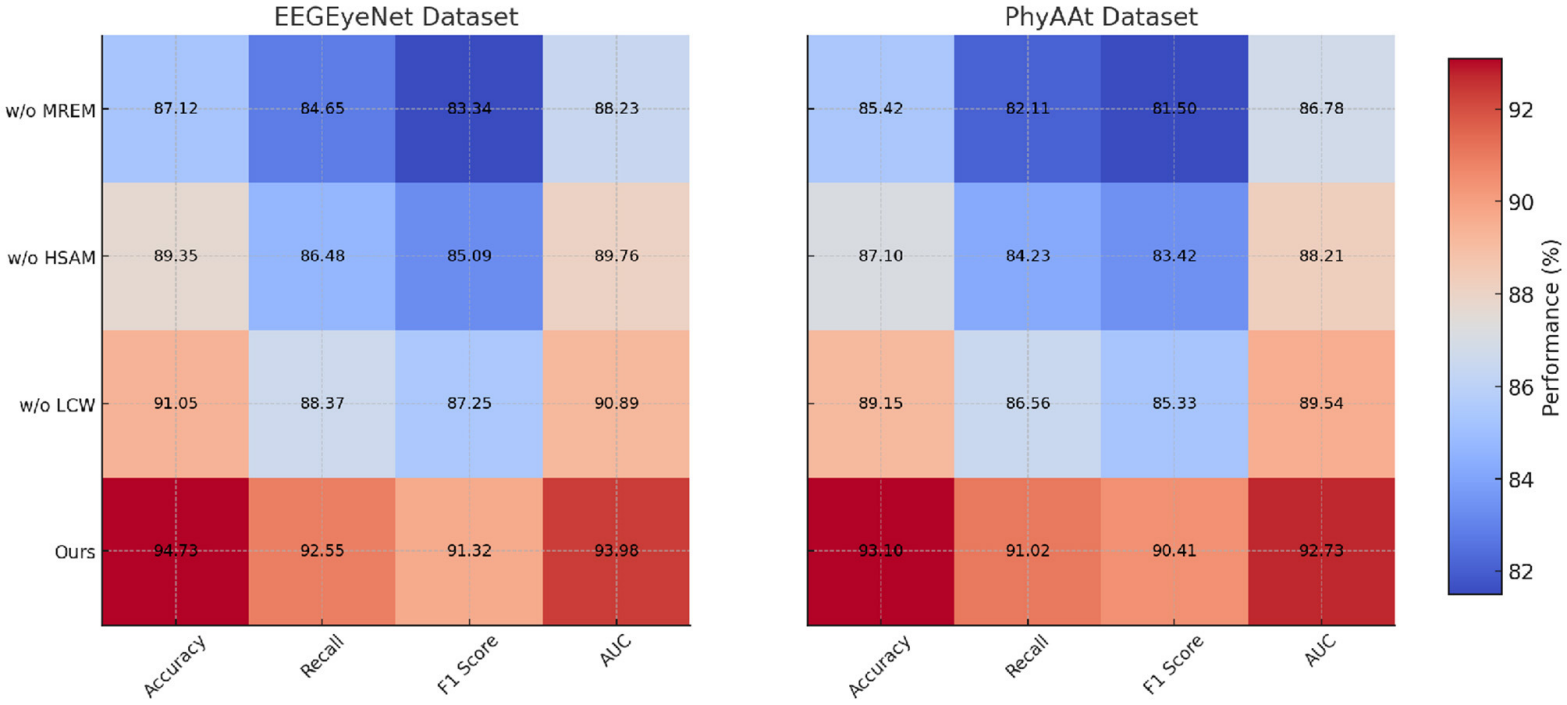
Ablation study of our method on EEGEyeNet dataset and PhyAAt dataset (MREM, multi-resolution embedding module; HSAM, hierarchical self-attention mechanism; LCW, localized context windowing).

Component Localized Context Windowing, associated with our model's multi-branch processing, was found to be particularly influential for PhyAAt and SEED datasets, which involve multi-dimensional sensor data and complex emotional EEG patterns, respectively. The removal of Component Localized Context Windowing led to marked declines in AUC values across datasets, demonstrating its effectiveness in refining feature extraction at different stages within the network (as shown in Figures 5, 6). On the PhyAAt dataset, AUC dropped from 92.73 to 89.54%, suggesting that this component enhances the model's ability to distinguish subtle variations in physical activities by separately processing each modality before integrating their outputs. The hierarchical feature extraction provided by Component Localized Context Windowing thus significantly boosts the model's capacity for detailed data analysis, particularly in tasks requiring nuanced recognition of physical movements or emotion-driven neural patterns.

In the experimental (in Table 5), we aimed to validate the performance of our model on multimodal emotion recognition tasks using two widely adopted datasets, IEMOCAP and EmotiCon. These datasets include various modalities such as text, speech, video, and EEG data, allowing us to thoroughly evaluate the model's capability in handling multimodal fusion tasks. The experimental setup involved preprocessing all modalities to ensure standardization, alignment, and feature extraction. Text embeddings were derived using BERT, speech features were extracted through wav2vec2.0, video features were obtained with ResNet, and EEG data were processed using convolutional neural networks to capture spectral characteristics. The experiments covered different modality combinations, ranging from single-modal text to multimodal setups such as text combined with speech, video, or EEG. Performance was assessed using accuracy, macro F1 score, weighted F1 score, precision, and recall. The results demonstrated the superiority of our EmotionFusion-Transformer in handling multimodal emotion recognition tasks. On the IEMOCAP dataset, the single-modal setup using text alone achieved an accuracy of 81.34% and a macro F1 score of 79.23%, highlighting the significant role of textual information in emotion recognition. On the EmotiCon dataset, the performance of text as a single modality was slightly lower, with an accuracy of 78.45% and a macro F1 score of 76.32%. With multimodal fusion, significant improvements were observed. Combining text, speech, and video modalities on the IEMOCAP dataset increased accuracy to 88.93% and macro F1 to 87.68%. Adding EEG data further elevated performance, achieving an accuracy of 91.45% and a macro F1 score of 90.12%, underscoring the complementary role of EEG signals in emotion recognition. Similarly, the EmotiCon dataset showed the highest performance with the full-modal setup, achieving an accuracy of 89.76% and a macro F1 score of 88.43%. These findings quantitatively demonstrate the advantage of multimodal inputs, as the integration of diverse modalities significantly enhances the model's ability to recognize emotions, with EEG data in particular contributing an additional 2.5% improvement in accuracy on IEMOCAP.

**Table 5:** Performance comparison of EmotionFusion-Transformer on IEMOCAP and EmotiCon datasets.

**Dataset**	**Modality**	**Accuracy (%)**	**Macro F1 (%)**	**Weighted F1 (%)**	**Precision (%)**	**Recall (%)**
IEMOCAP	Text only	81.34 ± 0.45	79.23 ± 0.32	80.45 ± 0.36	80.12 ± 0.50	78.89 ± 0.48
	Text + audio	85.76 ± 0.42	84.11 ± 0.33	84.65 ± 0.29	85.23 ± 0.40	83.45 ± 0.39
	Text + audio + video	88.93 ± 0.39	87.68 ± 0.28	88.21 ± 0.30	88.42 ± 0.33	87.11 ± 0.34
	All modalities (text + audio + video + EEG)	**91.45** **±0.35**	**90.12** **±0.30**	**90.78** **±0.31**	**91.03** **±0.28**	**89.87** **±0.29**
EmotiCon	Text only	78.45 ± 0.52	76.32 ± 0.40	77.01 ± 0.42	77.45 ± 0.50	75.98 ± 0.47
	Text + video	82.78 ± 0.48	81.12 ± 0.39	81.67 ± 0.35	82.11 ± 0.41	80.45 ± 0.36
	Text + video + audio	86.92 ± 0.45	85.65 ± 0.31	86.12 ± 0.38	86.42 ± 0.34	84.87 ± 0.33
	All modalities (text + video + audio + EEG)	**89.76** **±0.39**	**88.43** **±0.32**	**88.98** **±0.36**	**89.34** **±0.37**	**87.78** **±0.34**

Bold values are the best values.

## 5 Conclusions and future work

This study has demonstrated the effectiveness of the proposed EmotionFusion-Transformer framework in enhancing multimodal emotion recognition through the integration of EEG and textual data. By leveraging the complementary strengths of these modalities, the model achieves a nuanced understanding of emotional states, outperforming existing single- and multi-modality approaches in accuracy and robustness. The findings underline the potential of transformer-based architectures in capturing complex contextual dependencies and aligning multimodal data for improved performance. The experimental results on diverse datasets validate the adaptability of the framework across applications in emotion recognition, sleep stage classification, and eye-tracking tasks. However, the study acknowledges the challenges associated with the high-dimensionality of EEG features and their dependence on specific sensor configurations, which limits the practicality of direct implementation in consumer-grade devices.

An important future direction is to explore the critical EEG sensor locations from the current dataset to minimize the number of EEG features while maintaining classification accuracy at a usable level. Reducing the required sensor locations could significantly simplify hardware requirements and computational overhead, facilitating the transition of this research into practical applications. This investigation will involve systematic methods such as saliency map analysis and channel-wise ablation studies to identify the most impactful sensors. The results could inform the optimization of existing dry electrode headsets or guide the design of new, lightweight devices tailored for emotion recognition tasks. Such advancements would not only support the development of cost-effective and user-friendly systems but also bridge the gap between academic research and real-world implementations, fostering progress in areas such as affective computing, mental health monitoring, and human-computer interaction.

## Data Availability

The original contributions presented in the study are included in the article/supplementary material, further inquiries can be directed to the corresponding author.
